# Metastatic Tumour Burden as Prognostic Factor in Nivolumab-Treated Metastatic Renal Cell Carcinoma: An Exploratory Study

**DOI:** 10.3390/curroncol33060320

**Published:** 2026-05-28

**Authors:** Mario Uccello, Abigail L. Gee, James A. Bennett, Helen L. Hazell, Manuel Ruiz-Echarri Rueda, Mark J. Beresford

**Affiliations:** Royal United Hospitals Bath NHS Foundation Trust, Bath BA1 3NG, UK

**Keywords:** renal cell carcinoma, tumour burden, systemic inflammation, immunotherapy, nivolumab

## Abstract

**Introduction**: Prognostic assessment remains fundamental in metastatic renal cell carcinoma (mRCC). Established prognostic models, such as the International Metastatic Renal Cell Carcinoma Database Consortium (IMDC) and Meet-URO classifications, are widely used but do not directly quantify the metastatic tumour burden. We evaluated the prognostic value of metastatic tumour burden in nivolumab-treated mRCC and developed an exploratory composite prognostic score integrating the tumour burden with systemic inflammation and performance status. **Patients and Methods**: We retrospectively analysed consecutive patients with mRCC initiating nivolumab as a second- or third-line therapy at a single UK centre between March 2017 and November 2024. Their baseline clinical, laboratory and radiological data were collected. The metastatic tumour burden was defined as involvement of ≥2 metastatic organ sites, excluding lymph nodal, soft tissue, renal, pancreatic and thyroid metastases, while including bone metastases. The Bath score combined the metastatic tumour burden, systemic immune-inflammation index (SII) and Karnofsky performance status (KPS). Overall survival (OS) was analysed using Kaplan–Meier methods and a Cox regression, and the prognostic performance was compared using Harrell’s concordance index (C-index). **Results**: Fifty-one patients were included. After a median follow-up of 46.1 months, the median OS was 30.4 months. In the univariate analyses, a KPS < 80%, high SII, elevated platelet count and ≥2 metastatic organ sites were significantly associated with inferior OS. A KPS < 80% was the only variable retaining statistical significance in the multivariate analysis. The Bath score was significantly associated with OS (log-rank *p* < 0.001) and showed a numerically higher C-index than the IMDC and Meet-URO in this cohort (0.779 [optimism corrected, 0.760] vs. 0.641 and 0.706, respectively), with separation of risk groups. **Conclusions**: In this real-world cohort of nivolumab-treated mRCC, the metastatic tumour burden, systemic inflammation, and performance status were associated with survival. The Bath score should be regarded as exploratory and hypothesis-generating and requires validation in larger contemporary cohorts.

## Simple Summary

Kidney cancer that has spread to other parts of the body can behave very differently from one patient to another. Doctors use prediction tools to estimate how patients may do over time, but the commonly used tools do not directly measure how widely the cancer has spread across different organs. In this study, we reviewed 51 patients with advanced kidney cancer treated with nivolumab, an immunotherapy drug, at a single United Kingdom hospital. We explored whether the number of organs affected by cancer, blood markers of inflammation, and general fitness could help predict survival. Patients with cancer involving two or more organ sites, higher inflammation in the blood, or poorer general fitness tended to have shorter survival. We also developed an exploratory score combining these factors. This score appeared to separate the patients into different survival groups, but it was tested only in this small group of patients. Therefore, it should not be used in clinical practice without further validation. This study adds to growing evidence that the overall amount and spread of cancer may be important when estimating prognosis in advanced kidney cancer.

## 1. Introduction

Metastatic renal cell carcinoma (mRCC) remains a clinically challenging condition with substantial variation in outcomes, even in the era of contemporary systemic therapies. Immune checkpoint inhibitors (ICIs) have become an integral part of the management of mRCC, either alone or in combination with oral tyrosine kinase inhibitors (TKIs), and have significantly improved treatment outcomes [1]. Despite therapeutic advances, accurate prognostic stratification remains important in daily practice. Prognostic models use routinely collected clinical data to estimate survival risk, allowing clinicians to characterise the disease course, plan care, and inform treatment decisions. Real-world data play a valuable role in refining prognostic tools for mRCC, as they capture broader patient characteristics, patterns of metastatic spread, and treatment courses not always represented in clinical trials [2,3]. Several clinical tools have been proposed over time, including the Memorial Sloan-Kettering Cancer Center (MSKCC/Motzer) criteria, the International Metastatic Renal Cell Carcinoma Database Consortium (IMDC) model, and more recently the Meet-URO classification [4,5,6]. These models are widely used and clinically informative, but they do not incorporate a quantitative assessment of tumour burden. Indeed, growing clinical experience suggests that the extent and distribution of metastatic disease may influence the prognoses for patients receiving systemic anti-cancer therapy for mRCC [7,8,9]. Individuals with fewer visceral sites of involvement often present with slower disease kinetics and longer clinical benefit, whereas more extensive organ spread has been associated with shorter survival and earlier progression [10]. Systemic inflammation has also been recognised as a relevant prognostic dimension in mRCC. Notably, the IMDC model treats peripheral neutrophilia and thrombocytosis as separate adverse prognostic factors [5], although both reflect underlying systemic inflammation and are often correlated in mRCC populations. Analysing the correlated laboratory parameters as independent predictors may limit interpretability, whereas composite inflammatory indices, such as the neutrophil-to-lymphocyte ratio (NLR) and the systemic immune-inflammation index (SII), provide a unified framework for representing inflammatory status [7,11,12]. Performance status is also a recognised prognostic factor in mRCC and remains a routine component of clinical assessment [5,13]. In this retrospective study, we explored the prognostic relevance of metastatic burden in nivolumab-treated mRCC and evaluated an exploratory composite score, integrating organ dissemination with systemic inflammation and performance status using routinely available real-world data. This was motivated by the observation that the established models used in routine practice do not directly quantify metastatic dissemination or tumour burden. This analysis was designed to provide preliminary real-world evidence and to generate hypotheses for validation in larger cohorts.

## 2. Patients and Methods

We conducted a retrospective evaluation of consecutive patients with mRCC who received nivolumab as a second- or third-line systemic anti-cancer therapy at the Royal United Hospitals Bath NHS Foundation Trust. Adults with mRCC who initiated nivolumab between March 2017 and November 2024 were identified using electronic clinical and prescribing records. Eligible patients were those who had received at least one dose of nivolumab for previously treated mRCC and had available baseline clinical, laboratory, and radiological data at the time of treatment initiation. Patients treated with nivolumab as a first-line therapy or within clinical trials were excluded. The project was carried out as a retrospective service evaluation using anonymised data in accordance with local NHS governance procedures; formal research ethics review and individual patient consent were not required. Nivolumab administration reflected evolving UK regulatory approvals and NHS prescribing practice during the study period. Initially, patients received weight-based intravenous nivolumab every two weeks. After the flat-dose 480 mg every-four-weeks intravenous regimen became available in routine UK practice, all patients in this cohort were switched to the every-four-weeks fixed-dose schedule at the earliest clinically appropriate opportunity. A subcutaneous formulation of nivolumab became available in the UK in 2025, and in our centre patients on long-term intravenous nivolumab were progressively switched to subcutaneous administration from October 2025 onwards [14]. Nivolumab treatment was continued until disease progression, unacceptable toxicity, or patient or clinician decision. The baseline demographic and clinical information was extracted retrospectively from electronic oncology notes, laboratory systems, and radiology reports and entered into an anonymised database frozen on 30 November 2025. The variables recorded at nivolumab initiation included age, sex, histology subtype, Fuhrman nuclear grade, sarcomatoid differentiation, prior nephrectomy, number of previous systemic therapies, IMDC risk group, and Karnofsky performance status (KPS). Anthropometric and biochemical parameters were also collected, including height, weight, body mass index (BMI), serum creatinine, serum albumin, and haematological values. Systemic inflammation was quantified using the SII, calculated as platelet count × neutrophil count/lymphocyte count using baseline full blood count values [15]; the NLR was also calculated for exploratory purposes. The optimal SII cut-off for survival analysis within this cohort was derived using maximally selected rank statistics for overall survival (OS). Prognostic stratification was performed using the IMDC model, the Meet-URO classification, and an exploratory clinical score developed in this study, referred to as the Bath score. The IMDC and Meet-URO risk groups were determined according to their originally published criteria [5,6]. The Bath score integrated three routinely available prognostic dimensions: metastatic tumour burden, systemic inflammation as assessed by the SII, and performance status. The baseline cross-sectional imaging was defined as the scan performed closest prior to nivolumab initiation, preferably within 4–6 weeks of treatment start. Subsequent imaging was typically performed at approximately 3-month intervals or earlier if clinically indicated, in accordance with routine clinical practice. The metastatic tumour burden was defined a priori as involvement of two or more distinct organ sites on the baseline cross-sectional imaging obtained immediately before nivolumab initiation, reflecting a pragmatic clinical definition of multi-organ metastatic dissemination. The definition was informed by prior evidence showing prognostic heterogeneity according to the metastatic site in RCC. Lymph nodal, soft tissue, pancreatic, and thyroid metastases were excluded from the metastatic organ count owing to their relatively indolent clinical behaviour in mRCC [16]. This approach was intended to emphasise dissemination across the sites more consistently associated with adverse prognosis in RCC, while recognising that pancreatic and thyroid metastases may follow a more indolent course. By contrast, bone metastases were included given their established association with inferior outcomes [6]. Contralateral renal lesions were not counted as a visceral organ site because distinguishing renal metastasis from a metachronous, potentially indolent second primary tumour can be challenging and may require molecular or pathological correlation beyond imaging alone [17]. To improve reproducibility, the metastatic tumour burden was defined according to a prespecified site-based approach applied uniformly across the baseline imaging, rather than according to lesion size or volumetric measurements, which are less consistently available in routine retrospective practice. Performance status was assessed using the KPS, with a threshold of <80% considered adverse. Each adverse factor contributed one point, yielding a total Bath score ranging from 0 to 3, and patients were stratified into favourable- (0–1 adverse factors), intermediate- (2 adverse factors), and poor-risk (3 adverse factors) categories. The model was named the Bath score in reference to its origin in a single-centre UK cohort from the historical city of Bath. The clinical outcomes were collected retrospectively. OS was defined as the interval between nivolumab initiation and death from any cause or the last-documented follow-up. Progression-free survival (PFS) was defined as the interval between treatment initiation and radiological or clinical disease progression or death. The best radiological response was evaluated according to the Response Evaluation Criteria in Solid Tumours (RECIST), version 1.1; atypical response patterns with ICIs were interpreted in line with the immune RECIST (iRECIST) guidance [18,19]. Treatment-related toxicities were captured from clinical documentation, and the use of systemic corticosteroids initiated to manage immune-related adverse events was specifically recorded. The patients who were alive at database lock were censored at last follow-up. Survival curves were estimated using the Kaplan–Meier method. The associations between clinical variables and survival outcomes were explored using univariate log-rank tests. Prognostic models of interest, including the Bath score, IMDC risk groups, and Meet-URO classification, were analysed using a Cox proportional hazards regression to estimate the hazard ratios (HRs) with 95% confidence intervals (CIs). An exploratory multivariate Cox proportional hazards analysis for OS was constructed a priori to include the evaluable individual IMDC components together with the metastatic tumour burden and SII, representing the two non-KPS components of the Bath score. The variables were not selected using automated stepwise procedures. Neutrophilia and hypercalcaemia were excluded because of their very low frequency in this cohort. Bone metastases and elevated NLRs were not entered separately because they were considered to overlap clinically with the tumour burden and inflammatory domains captured by the Bath score. Harrell’s concordance index (C-index) was calculated to evaluate the discriminative ability of prognostic models. To quantify the potential optimism bias, internal validation was performed using 1000 bootstrap resamples. Within each bootstrap sample, the SII cut-off was first re-derived by selecting the threshold with the highest log-rank χ^2^ statistic; the full Bath score was then recalculated using this bootstrap-derived SII threshold together with the prespecified thresholds for metastatic burden (≥2 organ sites) and KPS (<80%), before fitting the Cox model and computing Harrell’s C-index. Statistical analyses were performed using MedCalc, version 23.5.2 (MedCalc Software Ltd., Ostend, Belgium), and R version 4.4.2 (R Foundation for Statistical Computing, Vienna, Austria) in RStudio version 2026.04.0+526 (Posit Software, PBC, Boston, MA, USA). Bootstrap validation and C-index calculation were performed in RStudio using the survival package. All statistical tests were two-sided, with significance set at *p* < 0.05.

## 3. Results

### 3.1. Baseline Characteristics

A total of 51 patients with mRCC who received nivolumab as a second- or third-line systemic therapy were included (Table 1). The median age at treatment initiation was 71 years (range, 41–81), and most were male (*n* = 40; 78.4%). A KPS ≥ 80% was observed in 30 patients (58.8%), while 21 (41.2%) had a KPS < 80%. Clear cell histology was the predominant subtype (*n* = 44; 86.3%), with papillary, unclassified or mixed variants occurring less frequently. Sarcomatoid differentiation was identified in eight cases (15.7%). A high proportion had undergone a prior nephrectomy (*n* = 45; 88.2%). Sunitinib was the most commonly used first-line TKI (*n* = 33; 64.7%), followed by pazopanib (*n* = 9; 17.6%), tivozanib (*n* = 4; 7.8%) and cabozantinib (*n* = 3; 5.9%). Nivolumab was administered as a second-line therapy in 41 patients (80.4%), with the remaining 10 receiving treatment in a third-line setting (19.6%). Lung and/or pleural metastases were the most frequent sites of distant disease (*n* = 36; 70.6%), followed by bone (*n* = 27; 52.9%), liver (*n* = 16; 31.4%), adrenal glands (*n* = 12; 23.5%) and pancreas (*n* = 8; 15.7%), with less common involvement of the brain, peritoneum or other organs.

### 3.2. Treatment Outcomes

The median follow-up at database freeze was 46.1 months (95% CI, 37.5–63.4). The median OS from nivolumab initiation was 30.4 months (95% CI, 16.3–39.7). The median PFS was 3.2 months (95% CI, 2.6–7.0). Complete responses were observed in five patients, with eight partial responses and 11 cases of stable disease, yielding an objective response rate of 25.5% and a disease control rate of 47.1%. Grade 3 immune-related adverse events occurred in four patients: insulin-dependent diabetes mellitus in two, immune-mediated pericarditis in one, and headache in one. Systemic corticosteroids were required in 14 patients (27.5%) to manage immune-related toxicities. Nivolumab was discontinued because of toxicity in three patients.

### 3.3. Prognostic Factors Associated with OS

In the univariate Cox proportional hazards analyses for OS (Table 2), KPS < 80%, high SII, elevated platelet count, and the presence of two or more metastatic sites were significantly associated with inferior OS (HR 4.45, 95% CI 2.14–9.25, *p* < 0.001; HR 3.98, 95% CI 1.91–8.32, *p* < 0.001; HR 2.96, 95% CI 1.02–8.58, *p* = 0.046; and HR 3.39, 95% CI 1.43–8.01, *p* = 0.005, respectively). An NLR ≥ 3.2 and the presence of bone metastases were associated with OS, although these associations did not reach conventional statistical significance (HR 1.98, 95% CI 0.99–3.94, *p* = 0.052; and HR 1.96, 95% CI 0.95–4.04, *p* = 0.070). The presence of lung and brain metastases was also significantly associated with inferior OS in the univariate analyses. Other variables, including age, sex, prior nephrectomy, sarcomatoid differentiation, liver or pancreatic metastases, BMI, haemoglobin level, time from diagnosis to first-line therapy, and first-line PFS, were not associated with OS in the univariate analyses. Corrected calcium and neutrophilia were not analysed because only one patient had hypercalcaemia and only two had neutrophilia at baseline, respectively.

### 3.4. Multivariate Analysis for OS

An exploratory multivariate Cox proportional hazards analysis for OS was performed, including the evaluable individual IMDC variables together with the metastatic tumour burden and SII (Table 3). As in the univariate analyses, neutrophilia and hypercalcaemia were not included because of their very low frequency in this cohort. In this model, KPS < 80% remained independently associated with inferior OS (HR 2.58, 95% CI 1.06–6.31, *p* = 0.037). The metastatic tumour burden and high SII showed a numerical association with inferior OS after adjustment, although these associations did not reach conventional statistical significance (HR 2.42, 95% CI 0.95–6.20, *p* = 0.064; and HR 2.22, 95% CI 0.93–5.32, *p* = 0.074, respectively). The time from diagnosis to first-line therapy < 1 year, thrombocytosis, and low haemoglobin were not independently associated with OS in this exploratory model.

### 3.5. Prognostic Performance of IMDC, Meet-URO and Bath Score

**Figure 1:**
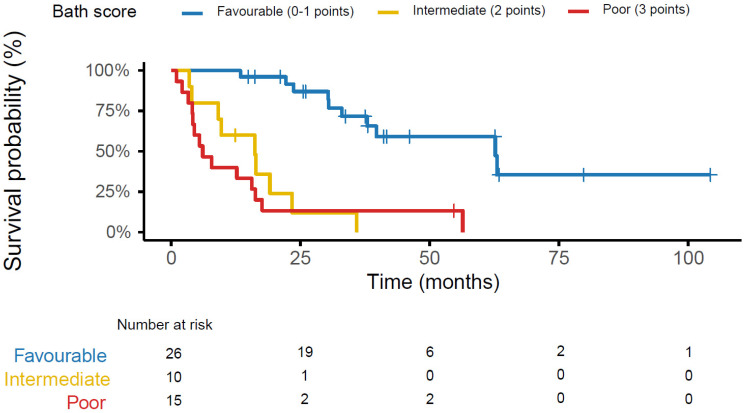
Kaplan–Meier overall survival curves according to the Bath score for patients with metastatic renal cell carcinoma treated with nivolumab. The numbers at risk are shown below the plot. The survival curves should be interpreted cautiously in the context of the small cohort size, although the overall survival follow-up was reasonably mature, with 33 deaths among 51 patients and 11 of 18 censored patients followed beyond the cohort median overall survival of 30.4 months.

The prognostic performances of the IMDC, Meet-URO and Bath score for OS were evaluated in patients treated with nivolumab (Table 4). All three prognostic models were significantly associated with OS by log-rank testing. According to the IMDC classification, OS differed significantly across risk groups (log-rank *p* = 0.006), with a Harrell’s C-index of 0.641 (95% CI 0.560–0.723). Using the Meet-URO classification, OS also differed significantly across risk categories (log-rank *p* < 0.001), with a Harrell’s C-index of 0.706 (0.628–0.785). The Bath score was significantly associated with OS across its risk categories (log-rank *p* < 0.001). The apparent Harrell’s C-index for the Bath score was 0.779 (95% CI 0.726–0.833). The bootstrap internal validation showed an optimism-corrected C-index of 0.760, corresponding to a mean estimated optimism of 0.019. The OS according to the Bath score is illustrated in Figure 1. The PFS also differed significantly across the Bath score categories (log-rank *p* = 0.031), with a median PFS of 5.8 months in the favourable-, 3.0 months in the intermediate-, and 2.5 months in the poor-risk groups. The ORR was 38.5%, 10.0% and 13.3% in the Bath favourable-, intermediate- and poor-risk groups, respectively, with no statistically significant difference across groups (*p* = 0.094).

## 4. Discussion

In this single-centre cohort of pretreated mRCC patients receiving nivolumab, the Bath score showed a numerically higher apparent discriminative performance for OS than the IMDC and Meet-URO (C-index 0.779 [optimism corrected 0.760] vs. 0.641 and 0.706), with separation of risk strata. These findings are consistent with the hypothesis that metastatic organ dissemination may contribute to prognosis during immunotherapy and may not be fully captured by established clinicopathological models. External data are increasingly reinforcing this concept. In a contemporary first-line nivolumab plus ipilimumab cohort, Oshima et al. [7] combined the baseline radiological tumour burden (RECIST sum of diameters; threshold 200 mm) with the NLR and identified groups with markedly different outcomes, including a median OS of 44.3 months vs. 6.1 months and a median PFS of 17.4 months vs. 4.1 months in the best versus worst combined strata. A post hoc analysis of the CLEAR trial [9] similarly reported outcomes using the baseline metastatic characteristics and baseline tumour size metrics for lenvatinib plus pembrolizumab versus sunitinib, underscoring that the baseline extent and distribution of disease remain prognostically informative in modern combination regimens. These studies support the prognostic relevance of tumour burden, although their burden metrics differ from our pragmatic organ-site count. In our study, the tumour burden component is intentionally pragmatic, based on counting distinct organ sites with prespecified exclusions for typically indolent sites; this information can usually be derived from standard radiology reports and may be more practical in routine care than the RECIST sum of diameters, which requires formal target lesion selection and measurement. Systemic inflammation is the second pillar of the Bath score, represented by the SII. The association of SII with outcomes of ICI-treated mRCC has been reported previously, including in nivolumab-treated populations and in cohorts receiving nivolumab plus ipilimumab [12,20]. In our cohort, a high SII remained one of the strongest predictors of inferior OS, consistent with the concept that baseline systemic inflammation captures tumour–host interactions that constrain the probability of durable benefit from PD-1/PD-L1 blockade [21]. Performance status is the third component, and its prognostic relevance in mRCC is long-established; in ICI-treated populations it plausibly reflects both the tumour-related symptom burden and broader frailty, and influences the likelihood of remaining clinically stable long enough to achieve durable immune-mediated control [22,23]. In our multivariate analysis including the Bath score components together with the assessable IMDC factors, an impaired performance status remained independently associated with inferior OS, whereas the metastatic tumour burden and SII retained non-significant associations with inferior OS after adjustment. These findings should be interpreted cautiously in view of the modest sample size and the exploratory nature of the analysis. Sarcomatoid differentiation showed a non-significant trend towards favourable outcomes in our cohort. Although this was likely influenced by the small number of patients, it is directionally consistent with the recognised activity of immune checkpoint blockade in sarcomatoid-feature RCC [24] and contrasts with the historically adverse implications of sarcomatoid histology in the pre-ICI era [2]. A direct comparison with the IMDC and Meet-URO clarifies what the Bath score may be capturing. The IMDC was derived and validated initially in populations treated predominantly with VEGF-targeted therapy and remains widely used because it is simple and externally validated [5,22], but it includes no direct measure of tumour burden or metastatic dissemination. In CheckMate 214, a multivariate analysis of the individual IMDC components suggested that, within the nivolumab plus ipilimumab arm, four of six factors (anaemia, neutrophilia, thrombocytosis, and time from diagnosis) were not prognostic for OS, whereas the KPS and corrected calcium remained prognostic [23]. This illustrates a concrete mechanism by which the prognostic weight of classical variables can shift under immunotherapy, and supports re-evaluation of model composition rather than repeated re-weighting of overlapping laboratory signals. The Meet-URO was developed specifically for pretreated patients receiving nivolumab and has shown improved stratification over IMDC in that setting, and has subsequently been evaluated in additional cohorts [6,25,26]. However, it remains centred on clinical and blood-based variables and does not incorporate a standardised radiology-derived measure of tumour burden. Its construction also introduced the NLR on top of IMDC, which already included blood-count-derived inflammatory signals, raising a legitimate concern about redundancy and interpretability. In our cohort, the non-monotonic OS pattern observed across the Meet-URO risk groups likely reflects sample-size limitations and further supports cautious interpretation of comparative model performance. Beyond clinical prognostic models, molecular and tissue-based biomarkers have also been investigated in RCC, including immune-related markers, treatment-resistance signatures, and integrated multi-omics approaches [27,28,29,30,31]. Although these approaches remain challenging to implement routinely in clinical practice, they are likely to become increasingly central to prognostic assessment and treatment selection in the future. The treatment context of our cohort is important for external validity. The tumour burden in this study was assessed at nivolumab initiation in previously treated patients and should therefore be interpreted in the context of later-line therapy rather than as a baseline measure of untreated metastatic disease. Nivolumab was delivered as a second- or third-line therapy across a long time window, whereas current practice has moved toward first-line ICI-based combinations with heterogeneous post-ICI pathways [3]. In addition, the high nephrectomy rate in our cohort likely reflects selection and historical treatment patterns and may be enriched in patients with a more favourable biology and lower burden at presentation. This is consistent with large real-world datasets of pretreated patients receiving nivolumab, in which a prior nephrectomy is common and is associated with more favourable baseline characteristics, including a better IMDC distribution, lower NLR, and fewer bone metastases, and with improved OS overall [32]. It is also consistent with the timing of UK access to first-line nivolumab plus ipilimumab for intermediate- and poor-risk disease, which entered NHS use via managed access routes in 2019 [33], meaning that afterwards the later-line nivolumab cohorts included a higher proportion of favourable-risk IMDC patients, many of whom were treated initially with VEGF TKI-based strategies rather than first-line dual ICI. Therefore, these findings limit the generalisability to unselected mRCC populations, including patients with non-clear cell histology, those managed without a prior nephrectomy, or contemporary first-line immunotherapy-treated cohorts. In our cohort, separation of OS curves by the Bath score (Figure 1) was driven primarily by the favourable group (0–1 adverse factors) versus the intermediate- and poor-risk groups (2–3 adverse factors), and the long-term responders to ICIs, described in trial and real-world populations, could accentuate such a dichotomous separation. The PFS also differed across the Bath score categories, whereas the ORR showed the same directional pattern without reaching statistical significance, likely reflecting the modest sample size. It is important to note that several limitations should temper the conclusions. This was a retrospective single-centre analysis with a modest sample size, and although the bootstrap internal validation suggested only modest optimism in the Bath score C-index, the internally derived SII threshold and exploratory nature of the model mean that external validation remains essential before it can be considered clinically applicable. Accordingly, the numerical C-index comparison with the IMDC and Meet-URO should be regarded as descriptive only and should not be interpreted as evidence of superiority of the Bath score over externally validated models. The exploratory multivariate analysis was also limited by the relatively small number of events for the number of covariates included. The metastatic sites were abstracted from contemporaneous radiology reports without a central review, and our burden definition captured organ dissemination rather than continuous volumetric metrics; both choices improve applicability in routine practice but are less radiologically accurate than quantitative volumetric methods. The nivolumab dosing schedules changed over time, which may have affected the monitoring frequency and the timing of treatment interruption or discontinuation and of response assessment in this retrospective cohort. Overall, these data should be regarded as exploratory and hypothesis-generating, and suggest that pragmatic assessment of visceral metastatic dissemination, together with systemic inflammation and performance status, may warrant further study as a prognostic approach in ICI-treated mRCC. The Bath score requires validation in larger contemporary cohorts before any conclusions can be drawn regarding its comparative prognostic utility.

## Figures and Tables

**Table 1:** Baseline characteristics of patients (*n* = 51).

Variable	Value
Age	
Median (range), years	71 (41–81)
Sex	
Male	40 (78.4)
Female	11 (21.6)
KPS	
≥80%	30 (58.8)
<80%	21 (41.2)
Histological subtype	
Clear cell RCC	44 (86.3)
Papillary RCC	3 (5.9)
Unclassified/mixed	4 (7.8)
Sarcomatoid differentiation	
Present	8 (15.7)
Absent/unknown	43 (84.3)
Previous nephrectomy	
Yes	45 (88.2)
No	6 (11.8)
First-line TKI	
Sunitinib	33 (64.7)
Pazopanib	9 (17.6)
Tivozanib	4 (7.8)
Cabozantinib	3 (5.9)
Other	2 (3.9)
Line of therapy at nivolumab initiation	
Second line	41 (80.4)
Third line	10 (19.6)
Site of distant metastasis	
Lung/pleura	36 (70.6)
Bone	27 (52.9)
Liver	16 (31.4)
Adrenal	12 (23.5)
Pancreatic	8 (15.7)
Brain	5 (9.8)
Peritoneal	4 (7.8)
Thyroid	2 (3.9)
Other	6 (11.8)
BMI	
Median (range)	27.5 (15.1–47.8)
Albumin (g/L)	
Median (range)	40 (24–51)

Abbreviations: BMI = body mass index; KPS = Karnofsky performance status; RCC = renal cell carcinoma; TKI = tyrosine kinase inhibitor.

**Table 2:** Univariate analyses for OS.

Variable	HR	95% CI	*p* Value
Sex (male vs. female)	1.63	0.67–3.96	0.281
Age (>70 years vs. ≤70 years)	1.38	0.69–2.75	0.362
BMI (<30 vs. ≥30)	2.70	0.81–8.99	0.105
KPS (<80 vs. ≥80)	4.45	2.14–9.25	<0.001
Prior nephrectomy (no vs. yes)	1.33	0.46–3.82	0.597
PFS1 (≥15 months vs. <15 months)	1.67	0.80–3.49	0.174
Prior lines of therapy (2 vs. 1)	0.94	0.42–2.12	0.880
Sarcomatoid component (yes vs. no)	0.47	0.16–1.39	0.171
Time from diagnosis to 1L (<1 year vs. ≥1 year)	1.34	0.66–2.73	0.416
Lung metastases (yes vs. no)	2.42	1.09–5.38	0.030
Liver metastases (yes vs. no)	1.37	0.64–2.91	0.414
Brain metastases (yes vs. no)	4.44	1.64–12.03	0.003
Bone metastases (yes vs. no)	1.96	0.95–4.04	0.070
Pancreatic metastases (yes vs. no)	0.95	0.39–2.30	0.906
Metastatic organ sites (≥2 vs. <2)	3.39	1.43–8.01	0.005
Platelet count > ULN (yes vs. no)	2.96	1.02–8.58	0.046
Haemoglobin level < LLN (yes vs. no)	1.84	0.92–3.68	0.085
NLR ≥ 3.2 (yes vs. no)	1.98	0.99–3.94	0.052
SII (≥675 vs. <675)	3.98	1.91–8.32	<0.001

Abbreviations: 1L = first-line therapy; BMI = body mass index; CI = confidence interval; HR = hazard ratio; KPS = Karnofsky performance status; LLN = lower limit of normal; NLR = neutrophil-to-lymphocyte ratio; OS = overall survival; PFS1 = progression-free survival on first-line therapy; SII = systemic immune-inflammation index; ULN = upper limit of normal.

**Table 3:** Multivariate analysis for OS.

Variable	HR	95% CI	*p* Value
KPS (<80 vs. ≥80)	2.58	1.06–6.31	0.037
Metastatic organ sites (≥2 vs. <2)	2.42	0.95–6.20	0.064
SII (≥675 vs. <675)	2.22	0.93–5.32	0.074
Time from diagnosis to 1L (<1 year vs. ≥1 year)	1.54	0.70–3.39	0.285
Platelet count > ULN (yes vs. no)	0.81	0.25–2.65	0.725
Haemoglobin level < LLN (yes vs. no)	1.10	0.50–2.39	0.817

Abbreviations: 1L = first-line therapy; CI = confidence interval; HR = hazard ratio; KPS = Karnofsky performance status; LLN = lower limit of normal; OS = overall survival; SII = systemic immune-inflammation index; ULN = upper limit of normal.

**Table 4:** Prognostic performance of IMDC, Meet-URO and Bath score for OS.

Model	Risk Group	*n* (%)	Deaths, *n*	mOS, mo (95% CI)	Log-Rank *p* Value	Harrell’s C-Index (95% CI)
IMDC risk	Favourable	18 (35.3)	10	37.9 (19.1–63.0)	0.006	0.641 (0.560–0.723)
	Intermediate	24 (47.1)	15	33.0 (12.7–62.7)		
	Poor	9 (17.6)	8	6.1 (2.1–16.4)		
Meet-URO	Risk 1	16 (31.4)	9	37.9 (19.1–63.0)	<0.001	0.706 (0.628–0.785)
	Risk 2	7 (13.7)	3	33.0 (22.2–33.0)		
	Risk 3	12 (23.5)	6	62.7 (9.1–62.7)		
	Risk 4	12 (23.5)	12	5.5 (2.1–16.4)		
	Risk 5	4 (7.8)	3	15.6 (4.1–16.2)		
Bath score	Favourable	26 (51.0)	11	62.7 (33.0–63.0)	<0.001	0.779 (0.726–0.833)
	Intermediate	10 (19.6)	9	16.2 (3.5–23.4)		
	Poor	15 (29.4)	13	6.1 (3.3–15.6)		

Abbreviations: IMDC = International Metastatic Renal Cell Carcinoma Database Consortium; Meet-URO = Metastatic Renal Cell Carcinoma–Meet the Urologists classification; OS = overall survival; mOS = median overall survival; CI = confidence interval; mo = months. Note: The Bath score C-index shown in the table represents the apparent C-index; the optimism-corrected C-index after the bootstrap internal validation was 0.760 and is reported in the text in the Results section.

## Data Availability

The original contributions presented in this study are included in the article. Further inquiries can be directed to the corresponding author.
